# Effects of the Heart to Heart Card Game for Patients with Advanced Cancer Receiving Home-Based Palliative Care: A Clinical Randomized Controlled Trial

**DOI:** 10.3390/ijerph19106115

**Published:** 2022-05-17

**Authors:** Jiayi Du, Ling Fu, Jiaxin Cui, Zifen An, Pei Fang, Lanhui Tan, Xianmei Meng, Liping Yu

**Affiliations:** 1 School of Nursing, Wuhan University, No. 115 Donghu Road, Wuhan 430071, China; jiayi.du@whu.edu.cn (J.D.); cjiaxin@whu.edu.cn (J.C.); zifen.an@whu.edu.cn (Z.A.); fangpei@whu.edu.cn (P.F.); tanlanhui@whu.edu.cn (L.T.); 2 Zhongnan Hospital of Wuhan University, No. 169 Donghu Road, Wuhan 430071, China; fuling@znhospital.cn

**Keywords:** cancer, Heart to Heart Card Game, palliative care, home care, quality of life, randomized controlled trial

## Abstract

The Heart to Heart Card Game improves psychological health outcomes in hospitalized patients with advanced cancer, but effectiveness studies for patients at home are rare. This randomized controlled study was conducted to determine the effectiveness of the Heart to Heart Card Game on patients with advanced cancer receiving home-based palliative care. Sixty-six participants were randomly assigned to the intervention group (*n* = 34) and control group (*n* = 32). The quality of life, dignity, and psychological distress were considered as outcomes, which were assessed pre-intervention and six weeks after the intervention. There was a statistical difference in the quality of life (global health statues) between the intervention group and the control group after intervention (*z* = 2.017, *p* < 0.05). A significant difference was found in the quality of life (emotional, social function), dignity (symptom distress dimension), and psychological distress in the intervention group through intragroup comparison before and after the intervention. This randomized trial showed that the Heart to Heart Card Game likely alleviates barriers to end-of-life conversations and helps patients with advanced cancer maintain a more stable mental state. This trial has been registered at the Chinese Clinical Trial Registry (registration number: ChiCTR2100049933).

## 1. Introduction

Cancer is becoming a critical health issue influencing quality of life (QoL), and an estimated 19.3 million new cancer cases occurred in 2020 worldwide [1,2]. The increasing incidence of cancer has substantially strained countries and caused overwhelming suffering for increasing numbers of patients and their families [2,3]. In particular, patients with advanced cancer have a poor Qol and experience psychological distress regardless of the type of cancer [4,5,6], indicating that interventions to improve QoL in this population are needed [7]. To provide appropriate care, the Institute of Medicine proposed patient-centered care, which highlights the vital role of patients’ values, needs, and preferences in clinical decision-making [8,9]. Understanding cancer patients’ preferences is recognized as an essential component in providing effective care to meet their needs and improve their outcomes [10,11,12].

Current studies have been concerned with the importance of cancer patients’ preferences in providing care [13,14]. Many studies have investigated cancer patients’ preferences. Ellis et al. [15] and Saracino et al. [16] found that most cancer patients preferred receiving as much information about cancer, treatments, costs, and expected outcomes as possible. Some researchers have explored the factors influencing cancer patients’ preferences [17,18]. Yilmaz et al. [18] found that Latin-American and African-American patients preferred shared or active participation while Asian and Middle-Eastern ethnic minority patients preferred passive participation during the decision-making process. A few studies have examined the effects of interventions in terms of patient preferences. Petzel et al. [14] conducted a web-based intervention in a randomized controlled trial to improve advanced care planning in women with ovarian cancer, and found that patients who received information (on topics including distress, coping, and stress management) based on their preferences were less distressed than those who did not. Delgado-Guay et al. [19] used the Go Wish card game to talk to patients with advanced cancer about their end-of-life preferences and found that more than half of the participants agreed that the game was beneficial.

Due to the Chinese ‘taboo death’ culture [20], communicating with individuals with advanced cancer and knowing their preferences poses more barriers and concerns for Chinese medical workers [21,22]. Thus, the Chinese American Coalition for Compassionate Care developed the Heart to Heart Card Game (HHCG), based on Go Wish cards, to help Chinese healthcare providers to initiate end-of-life conversations (see https://caccc-usa.org/en/activities/heart2heart.html for more details, accessed on 12 December 2021).

Li et al. [23] used the HHCG to investigate the preferences of Chinese cancer patients, and found that more than 70% of the participants highly valued the HHCG. Liu et al. [24] used the HHCG on 17 patients with malignant tumors in the oncology department, and compared them with their counterparts who received usual care. The results showed that palliative care guided by the HHCG can relieve cancer patients’ anxiety and depression, and improve their sense of dignity. He et al. [25] also found that the HHCG can improve the death-related attitudes of hospitalized patients with advanced cancer and effectively reduce their negative emotions of anxiety and depression. However, in the Eastern culture, patients with advanced cancer are more likely to receive palliative care at home [26]. The effects of the HHCG for patients with advanced cancer receiving home-based palliative care must therefore be explored.

Overall, the HHCG is a simple and easy-to-use tool suitable for Chinese cultural backgrounds and palliative care guided by the HHCG is beneficial for hospitalized cancer patients. However, many people with a terminal illness would prefer to receive end-of-life care at home [27]. There are limited studies evaluating the effects of the HHCG on patients with advanced cancer receiving home-based palliative care. Therefore, this study aimed to evaluate the effects of the HHCG on QoL, dignity, and psychological distress for patients with advanced cancer receiving home-based palliative care.

## 2. Conceptual Framework

This study is guided by the Sunrise Model developed by Leininger [28]. According to this model, the social structure and worldview components influence the folk, professional, and nursing system(s) [29], and three kinds of nursing care, decisions, and actions are predicted in the model: culture care preservation or maintenance, culture care accommodation or negotiation, and culture care repatterning or restructuring. Culture care preservation or maintenance refers to actions and decisions that help people to maintain meaningful care values and lifeways for their health-related outcomes. Cultural adaptation refers to the actions and decisions that help people to negotiate with others for meaningful, beneficial, and congruent health outcomes. Culture care repatterning or restructuring refers to the actions and decisions that help clients change or modify their lifeways for new, different, and beneficial health outcomes [28,29]. In this study, the HHCG was adjusted according to the Sunrise Model. The patients’ cultural backgrounds (e.g., religion, economics, cultural values) and preferences were ascertained by asking the participants to choose the important cards and having a semi-structured end-of-life conversation, and then the patient’s palliative care and family care were adjusted or maintained according to the patient’s sharing results. Based on the literature review and previous practical experience, Qol, dignity, and psychological distress were selected as the evaluation variables [28,30].

## 3. Materials and Methods

### 3.1. Study Design

This study was a 1:1 parallel design single-blinded clinical randomized controlled trial (RCT), with a study population consisting of advanced cancer patients receiving home-based palliative care. Additionally, this study’s design, conduction, and reporting adhere to the Consolidated Standards of Reporting Trials (CONSORT) guidelines.

### 3.2. Setting and Participants

This study was carried out from January 2021 to September 2021 at a Hospice Unit in Wuhan, China. The Hospice Unit provides home-based palliative care services within 100 km of Wuhan. The participant eligibility criteria were as follows: (1) older than 18 years, with a diagnosis of advanced cancer based on pathology (cancer in stage IV or stage III); (2) attending home-based palliative care services provided by the Hospice Unit; (3) physician-estimated life expectancy >3 months; and (4) Chinese speaking. Patients were excluded if they could not complete the HHCG independently because of physical symptom distress or physical limitations (e.g., visual or motor impairment).

### 3.3. Procedure

Randomization was carried out via an internet-based randomization program (www.cnstat.org/randomization/simple-sampling, accessed on 24 September 2020). The random allocation sequence was in a uniform 1:1 allocation ratio, and was concealed from patients and investigators assessing outcomes through sealed envelopes containing random number cards.

After obtaining patients’ informed consent, the evaluators collected participants’ demographic and psychological data. Then, the two researchers conducted the first home visits, confirmed which group the patient belonged to through the envelopes, and then carried out the corresponding intervention.

**Figure 1:**
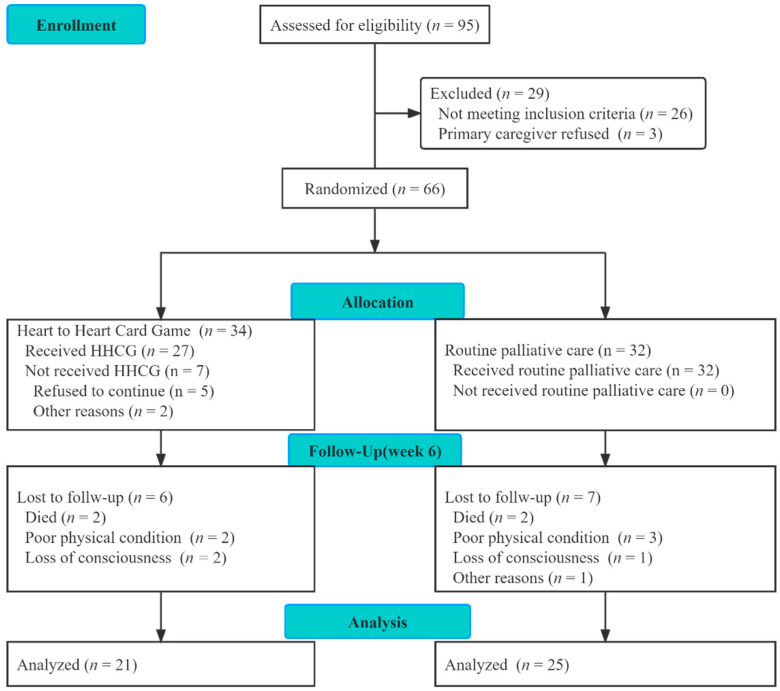
Participant flowchart of the RCT.

Both groups were evaluated by measurements prior to and six weeks after the intervention by the same investigators. Psychological data were collected at the patients’ homes through scales while the general demographic information of the patients was obtained via self-made questionnaires through the medical system of the Hospice Unit. Recruitment and enrollment of the participants is described in a trial flow diagram in Figure 1.

### 3.4. Intervention

#### 3.4.1. Control Group

The control group received routine palliative care provided by a trained interdisciplinary palliative care team composed of six nurses, two doctors, a social worker, and some volunteers. Routine palliative care included pain and symptom management, dietary and activity guidance, psychological support, a 24-h online consulting service, and 2 home visits. The palliative care team was divided into 2 groups to provide home visits to patients with advanced cancer, and 24-h online consultation was provided by nurses who did not participate in the home visits.

#### 3.4.2. Intervention Group

In addition to the same routine palliative care services, the participants in the intervention group received the HHCG at their first home visit. Two first authors, who had completed full training on the HHCG intervention, implemented all HHCGs in the patients’ homes. Considering the game’s peculiarities (talking about end-of-life preferences) and the survival time of patients with advanced cancer, we only conducted the intervention once. No additional intervention was applied to the two groups during the study period.

#### 3.4.3. Heart to Heart Card Game Intervention

Each deck of the Heart to Heart Cards contains 54 cards, including 13 Spades, 13 Hearts, 13 Diamonds, 13 Clubs, and 2 Jokers (Special Wish cards). Each of the four suits represents a particular kind of preference: Hearts for spiritual, Diamonds for financial, Clubs for social, and Spades for physical. In particular, each card represents an individual end-of-life preference. For example, the Heart A is “I want to maintain my dignity”. Additionally, the ‘Special Wish’ cards are blank, and allow patients to add two preferences not included in the other cards.

There were four steps in the HHCG intervention. In the first step of preparation and introduction, we created a relatively private and quiet space and introduced the content of Heart to Heart Cards and the rules of the game to the participants. After fully understanding the information, participants confirmed whether they wished to participate in the game or not. In the second step, choosing cards, participants were instructed to pick the 12 most essential cards from a deck of 54, followed by choosing the 3 most important cards from those 12 cards. In the third step, end-of-life conversation, the researcher conducted a semi-structured end-of-life conversation based on the cards selected by the participants, to understand their reasons for choosing the cards, the stories behind the cards, and the preferences and needs of the participants. Then, the investigators made a wish list according to the conversation and, finally, confirmed the information with the participants. The fourth step was information translation—conveying the patients’ preferences and needs to their patients’ families, making corresponding nursing plans, and providing support. The end-of-life conversation in the HHCG was semi-structured under a set of guidelines (Supplementary File S1). HHCG interventions ranged from 50 to 90 min, and varied with the participants’ conditions. We attached a whole intervention process as an example case (Supplementary File S2).

### 3.5. Tools

According to the results of Li et al. [23] “I want to maintain my dignity” and “I don’t want to suffer” were found to be the important preferences in HHCG among Chinese cancer patients. Therefore, we considered dignity and psychological distress as outcome variables. Moreover, the QoL is essential for patients with advanced cancer as shown in the literature review and was included as an outcome.

#### 3.5.1. Patient Information Form

Demographics were assessed using a self-designed questionnaire that was informed by the literature review. Since the patients served by the Hospice Unit were all low-income patients, the demographic data did not consider the patients’ financial status. The activities of daily living were assessed by the modified Barthel index (MBI), which represented the disability level of patients with advanced cancer [31]. A score of <60 indicates that the patient needs help to maintain daily life while 60–100 indicates that the patient can perform daily life independently [31].

#### 3.5.2. Quality of Life

We used the Chinese version of the European Organization for Research and Treatment of Cancer questionnaire entitled Quality of Life Questionnaire version 30 (EORTC-QLQ-C30) to evaluate the participants’ QoL. The EORTC-QLQ-C30 is a 30-item scale composed of 5 functional scales (physical, daily activities, emotional, cognitive, and social function), 3 symptom scales, a global health status scale, and 6 independent items about symptoms. Our research only considered functional scales (4-point Likert-type scale, ranging from “not at all” to “very much”) and global health status scales (7 points). A higher score indicated greater functionality and better QoL [32].

#### 3.5.3. Dignity

The Patient Dignity Inventory was administered to measure the primary outcome dignity. This is a 25-item assessment tool with 5 dimensions: symptom distress, existential distress, dependency, social support, and peace of mind. Each item is scored from 1 to 5 points (from “not a problem” to “an overwhelming problem”); the higher the score, the more serious the loss of dignity. The internal consistency reliability of the scale’s Cronbach’s α is 0.93 [33].

#### 3.5.4. Psychological Distress

The Distress Thermometer is recommended by the National Comprehensive Cancer Network, and can measure psychological distress [34]. It asks patients to rate their distress over the past week, including the present day, on a scale of 0 (no distress) to 10 (extreme distress) [35].

### 3.6. Statistical Methods

The collected data were analyzed using IBM SPSS Statistics 25 (IBM Corp. Released 2017. IBM SPSS Statistics for Windows, Version 25.0., IBM Corp.: Armonk, NY, USA). Continuous variables were described using means and standard deviations (SDs), or medians (quartiles) while categorical variables were described with absolute values and percentages (%). The normality of data was tested using the Shapiro–Wilk test. Independent sample *t*-tests, Fisher’s exact tests, and paired sample *t*-tests were used to detect the differences between the intervention group and the control group, or the pre-test and post-test differences within each group if the data conformed to the normal distribution. Otherwise, non-parametric tests were used. Missing values of the demographic data were imputed by mode. A conventional criterion of statistical significance (*p* < 0.05) was applied for all analyses.

### 3.7. Ethical Considerations

This study was approved by the Ethics Committee of Wuhan University (approval number: 2020YF0081), and was registered at clinicaltrials.gov accessed on 14 August 2021 (registration number: ChiCTR2100049933). The authors explained the study to all the participants, and they all signed informed consent before completing the baseline assessment. Participants could withdraw from the study at any time.

## 4. Results

### 4.1. Participants’ Characteristics

Sixty-six patients were eligible for participation and were randomized to the intervention (*n* = 34) and control (*n* = 32) groups and assessed over 6 weeks, yielding 21 patients for the HHCG group and 25 for the control group, with an attrition rate of 30%. Most of the participants were married and elderly, and the most common caregivers were their spouses. There were no differences between the HHCG and control groups in terms of demographic variables, QoL (function and global health status), dignity, and psychological distress scores at baseline (Table 1 and Table 2).

In our study, 79.4% of the participants in the intervention group completed the HHCG. One refused to continue because the participant thought that the HHCG was meaningless. Two participants thought that the HHCG was meaningful and were willing to participate in it in the future. Two cases refused due to family reasons, and two cases were discontinued due to objective reasons (e.g., needing to attend an appointment).

### 4.2. Card Selection Result in the Intervention Group

When choosing the 12 Heart to Heart cards, the most frequent choice of the participants was “I don’t want to be a burden to my family” (*n* = 21), followed by “I want to maintain my dignity” (*n* = 16), and then “I want my family to get along” (*n* = 15). Among the three most important cards selected, the patients’ most frequent choice was “I don’t want to be a burden to my family” (*n* = 10), followed by “I don’t want to suffer” (*n* = 6), and “If I’m going to die anyway, I don’t want to be kept alive by machines” (*n* = 6). Moreover, among the three cards selected, physical cards (47.9%) were rated as the most important, followed by social (28.2%), spiritual (12.7%), and financial (11.3%). Table 3 presents the top 5 preferences among the 12 cards and the 3 most important cards selected.

### 4.3. Effectiveness of HHCG

#### 4.3.1. Effect on Quality of Life

Statistical differences were found in the global health status scores of the intervention group and the control group after the intervention (*z* = 2.017, *p* = 0.044) (Table 2). The analysis of the pre- and post-intervention scores analysis only revealed a statistically significant difference in the emotional function and social function scores in the intervention group (*t* = 3.102, *p* = 0.006; *t* = 3.347, *p* = 0.003, respectively) (Table 4).

#### 4.3.2. Effect on Dignity

After the intervention, the dignity scores in the intervention group were lower than those of the control group. However, there were no statistically significant differences in the scores of the two groups (*z* = 0.077, *p* = 0.943). The reduction in the scores of the intervention group was higher than that in the control group (*z* = 2.470, *p* = 0.013).

The pre- and post-intervention scores revealed statistical reductions in the total dignity, existential distress, and peace of mind dimensions. However, no statistically significant differences were found in the dependency and social support dimensions in either the intervention or the control group (Table 4). A statistical difference in the dimensions of symptom distress only appeared in the HHCG group (*t* = 3.501, *p* = 0.002).

#### 4.3.3. Effect on Psychological Distress

No statistical differences were found in the psychological distress scores between the intervention group and the control group after the intervention (Table 2). In the paired analysis of psychological distress scores, only the scores of the intervention group were statistically reduced while those of the control group were not (*z* = 2.686, *p* = 0.014; *t* = 1.127, *p* = 0.271, respectively) (Table 4).

## 5. Discussion

In this study, we performed the HHCG on patients with advanced cancer receiving home-based palliative care, and our results suggest that the HHCG can maintain the patients’ psychological standards throughout the development of their disease. This study may provide a new approach to developing interventions aimed at providing psychological support for people with advanced cancer at home.

In our study, we included 66 participants, but only 46 were included in the final analysis—an attrition rate of 30%, which was strongly associated with participants being at the end of their lives. In addition, the attrition rate was higher in the intervention group (38%) than in the control group (21%), which was related to the rejection of the HHCG by the intervention group. However, 79.4% of participants in the intervention group completed the HHCG. Compared with a previous similar intervention (the Go Wish Card game), the completion rate of the intervention group in this study was not low [19,36]. In a previous study, 62% of the participants tended to complete the Go Wish Card games [19]. These differences might be related to the context in which we intervened and conducted the one-to-one approach.

The most frequently selected cards were the same as those in a previous study [23]. “I don’t want to be a burden to my family” was chosen the most often in this study. These results may be because patients with advanced cancer who receive home-based palliative care are primarily dependent on their family caregivers, which makes them feel like they are more of a burden [37,38]. Moreover, the cards with the highest frequency among the three most important cards were physical, suggesting that patients with advanced cancer who receive home-based palliative care are more concerned with their physical symptoms, which needs to be confirmed in further studies with a lager sample size.

Our study’s first major finding is that the HHCG can help patients with advanced cancer to improve their total dignity score, showing better results in this metric than the control group. The results were consistent with those of Liu et al. [24], who performed the HHCG in hospitalized cancer patients. The HHCG helps adults with advanced cancer to facilitate the expression of thoughts, feelings, and memories, which can help to improve their sense of dignity [39]. In addition, we found that the existential distress and peace of mind dimensions of the Patient Dignity Inventory improved among participants in both groups. Existential distress denotes the distress caused by the pressure of survival, such as a financial burden [33]. It is indisputable that cancer brings economic burden to patients and their families [40]. Most patients with advanced cancer have feelings of becoming burdensome [41]. The improvement in existential distress may have been due to the free painkillers provided by the Hospice Unit, which relieved the patients’ financial burden to a certain extent. Peace of mind concerns refers to the problems caused by things that patients have left undone and thoughts that their lives have contributed nothing [33]. The improvement in this dimension may have been related to the samples in the Hospice Unit having accepted the fact of their impending death. The family members providing home-based palliative care reported that they had to “face reality” to better enjoy their remaining time with their relatives [42]. Thus, the family members might also have looked for ways to satisfy the patients’ wishes in palliative care to improve the patients’ peace of mind.

Our study’s second main finding is that the HHCG can help patients to maintain a better Qol. Bouleuc et al. [43] conducted a trial in French using a Question Prompt List for patients with advanced cancer to promote advance care planning but did not find a significant improvement in QoL. Compared to the Question Prompt List, HHCG is not only a useful tool to define the issues but also a one-to-one and patient-centered intervention that can help patients express their thoughts and emotions and receive timely feedback, which is likely related to the fact that the HHCG can respond to and meet the needs of patients with advanced cancer to maintain their Qol [43,44,45]. However, this improvement is limited to physical function. For patients with advanced cancer, the physical demands, such as not having dyspnea due to the deterioration of their physical condition being irreversible, are challenging to achieve [46].

Our study’s third major finding is that the HHCG can be beneficial in psychological distress. Previous studies have found that card games can reduce anxiety and depression in hospitalized patients [19,24,25]. Since psychological distress can indicate anxiety and depression, our results are consistent with previous results [47]. Among the interventions used to improve psychological distress, meaning-centered intervention, which aims to promote meaningful lives for cancer patients, has been suggested to improve psychological distress significantly [48,49]. The HHCG guides nurses and patients in making future arrangements according to the patients’ preferences, and may also enhance the patients’ sense of meaning in life.

Interestingly, we found that the social function scale of QoL of the HHCG group showed statistically significant differences while the social support dimension of the Patient Dignity Inventory did not. This inconsistency may be related to differences in the focus of the items on the two scales. The social function scale of QoL focuses on the physical condition or medical treatment that impacts patients’ family life and social activities [50]. This result may be related to the HHCG helping patients to adjust some aspects of daily life, such as going out for a walk. The social support dimension of the Patient Dignity Inventory places extra emphasis on the support and understanding perceived by the patient from family and health providers [33]. A possible explanation is that the mean score for social support was lower, indicating that the support and understanding perceived by our participants were at a normal level while the improvement effect of the HHCG on the social support dimension of the Patient Dignity Inventory was not noticeable.

## 6. Limitations

The present study also has some limitations. Firstly, considering the patients’ survival time and the inconvenience of home visits during Chinese New Year, we performed the HHCG only once during the intervention process, and the evaluation was also only carried out once six weeks after the intervention. Future studies could increase the frequency of interventions and evaluation to explore the long-term and short-term effects of the HHCG. Secondly, a number of the potential participants refused to participate since they did not want to talk about their last wishes. This may have resulted in some bias being present in the results, and is also a direction in which the HHCG needs to be improved in the future. Finally, we recruited patients from only one institution, and the sample size of our research was limited due to epidemic control. A larger multi-center research sample and a cost-benefit analysis are needed in the future.

## 7. Conclusions

This study conducted a single-blinded randomized controlled trial to examine the effects of the HHCG on QoL, dignity, and psychological distress in patients with advanced cancer who are receiving home-based palliative care. Due to the limited sample size, the results showing that the HHCG can improve the sense of dignity, psychological distress, and QoL still need to be confirmed by further studies with larger samples. However, our study expands the knowledge about the effects of the HHCG in patients with advanced cancer who are receiving home-based palliative care.

## Figures and Tables

**Table 1:** Demographic and clinical characteristics (*N* = 66).

Variables	HHCG (*n* = 34)		Control (*n* = 32)		*z*/Fisher’s Exact Test	*p*
	*n*	%	*n*	%		
Age (years)					1.189	0.277
<60	10	29.4	6	18.8		
60–80	21	61.8	21	65.6		
>80	3	8.8	5	15.6		
Gender					0.210	1.000
Male	20	58.8	18	43.8		
Female	14	41.2	14	56.2		
Marital status					0.186	1.000
Single	7	20.6	6	18.8		
Married	27	79.4	26	81.2		
Education					0.238	0.802
<6 years	14	41.2	9	28.1		
7–12 years	8	23.5	12	37.5		
13–15 years	4	11.8	7	21.9		
Higher	8	23.5	4	12.5		
Religion					1.040	0.348
No	33	97.1	29	90.6		
Yes	1	2.9	3	9.4		
Diagnostic location					0.360	0.727
Reproductive	7	20.6	9	28.1		
Digestive	11	32.4	9	28.1		
Respiratory	12	35.2	9	28.1		
Urinary	2	5.9	4	12.5		
Other	2	5.9	1	3.2		
Period (days)					0.986	0.326
<30	11	32.4	9	28.1		
30–90	7	20.6	20	62.5		
>90	16	47.0	3	9.4		
ADLs (MBI)					1.165	0.297
<60	31	91.2	26	81.2		
60–100	3	8.8	6	18.8		
Living condition					0.977	0.547
Relatives	32	94.1	31	96.9		
Other	2	5.9	1	3.1		
Primary caregivers					0.242	0.817
Spouse	24	70.6	21	65.6		
Children	5	14.7	7	21.9		
Other	5	14.7	4	12.5		

Abbreviations: ADLs = activities of daily living, <60 means dependence, 61–100 means independence; Period = days from entering the Hospice Unit to the first data collection; MBI = modified Barthel index.

**Table 2:** Effects of the Heart to Heart Card Games on dignity, psychological distress, and quality of life in the intervention and control group at the 6-week follow-up (*n* = 46).

Variables	Group	Baseline		*t* (*p*)/*z* (*p*)	Post-Test		*z* (*p*)
		Mean/Median	SD/P25, P75		Median	P25, P75	
**Quality of life**							
Physical	HHCG	33.33	10.00, 60.00	0.548 (0.591) ^b^	33.33	0.00, 76.67	0.672 (0.509)
	Control	33.33	0.00, 60.00		26.67	0.00, 56.67	
Role	HHCG	50.00	33.33, 83.33	0.202 (0.846) ^b^	66.67	8.33, 83.33	0.505 (0.620)
	Control	50.00	8.33, 83.33		33.33	16.67, 66.67	
Emotional	HHCG	66.67	33.33, 79.17	1.743 (0.084) ^b^	75.00	58.33, 100.00	0.458 (0.654)
	Control	83.33	50.00, 100.00		83.33	66.67, 91.76	
Cognitive	HHCG	83.33	66.67, 100.00	0.649 (0.528) ^b^	66.67	50.00, 83.33	0.476 (0.641)
	Control	83.33	58.33, 83.33		66.67	58.33, 83.33	
Social	HHCG	33.33	0.00, 50.00	1.264 (0.209) ^b^	50.00	33.33, 83.33	0.605 (0.554)
	Control	50.00	16.67, 66.67		50.00	33.33, 66.67	
Global health status	HHCG	66.67	58.33, 91.67	1.836 (0.067) ^b^	66.67	58.33, 91.67	2.017 (0.044) *
	Control	58.33	50.00, 75.00		50.00	50.00, 75.00	
**PDI (total)**	HHCG	62.14	19.03	1.526 (0.134) ^a^	39.00	29.50, 64.50	0.077 (0.943)
	Control	53.08	20.88		40.00	29.50, 62.50	
Symptom distress	HHCG	16.52	5.11	1.837 (0.730) ^a^	12.00	7.50, 18.50	0.377 (0.713)
	Control	13.72	5.20		10.00	7.00, 16.00	
Existential distress	HHCG	14.00	10.50, 24.00	1.791 (0.074) ^b^	8.00	6.00, 15.50	0.401 (0.695)
	Control	11.00	8.00, 16.00		10.00	7.00, 15.50	
Dependency	HCCG	5.00	3.50, 7.50	0.246 (0.812) ^b^	5.00	3.00, 7.00	0.722 (0.478)
	Control	5.00	3.50, 7.50		5.00	3.00, 8.50	
Social support	HCCG	5.00	3.50, 7.50	0.572 (0.575) ^b^	4.00	3.00, 7.00	0.189 (0.856)
	Control	5.00	3.00, 7.00		3.00	3.00, 7.00	
Peace of mind	HHCG	6.19	2.16	0.740 (0.410) ^a^	4.00	3.00, 6.00	0.068 (0.949)
	Control	6.24	2.35		5.00	3.00, 6.00	
**Psychological distress**	HHCG	5.00	3.00, 7.50	1.264 (0.209) ^b^	4.00	3.50, 5.00	0.123 (0.907)
	Control	4.00	3.00, 6.00		4.00	3.00, 6.00	

Abbreviations: HHCG = Heart to Heart Card Game. PDI = Score on the Patient Dignity Inventory. ^a^ Independent sample *t* test. ^b^ Mann–Whitney U. * *p* < 0.05.

**Table 3:** Card selection result in the HHCG group (*N* = 27).

Frequency of Top 3 Items	*n* (%)
**Among the 12 Cards Selected**	
I don’t want to be a burden to my family.	21 (77.8)
I want to maintain my dignity.	16 (59.3)
I want my family to get along.	15 (55.6)
I don’t want to suffer.	12 (44.4)
If I’m going to die anyway, I don’t want to be kept alive by machines.	12 (44.4)
I want to go outside.	12 (44.4)
I want my family to remember the happy times.	12 (44.4)
**Among the 3 Most Important Cards Selected**	
I don’t want to be a burden to my family.	10 (37.0)
I don’t want to suffer.	6 (22.2)
If I’m going to die anyway, I don’t want to be kept alive by machines.	6 (22.2)
I want to maintain my dignity.	5 (18.5)
I don’t want to suffer from shortness of breath.	5 (18.5)
I want to know how what’s going to happen next.	5 (18.5)

**Table 4:** Evaluation of Heart to Heart Card Games on quality of life, dignity, and psychological distress in the intervention and control group (*N* = 46).

Variables	Group	Paired *t*-Test/Wilcoxon Signed-Rank Test		Mean/Median Difference	*t*/*z*	*p*
		*t*/*z*	*p*	M (95% CI)		
**Quality of life**						
Physical	HHCG	0.481	0.481 ^a^	0.00 (−6.67, 6.67)	0.506	0.506 ^d^
	Control	1.565	0.157 ^b^			
Role	HHCG	0.556	0.584 ^a^	0.00 (0.00, 16.67)	0.143	0.892 ^d^
	Control	1.512	0.201 ^b^			
Emotional	HHCG	3.102	0.006 ^a,^*	−3.20 (−6.88, 0.48)	1.774	0.086 ^c^
	Control	1.919	0.067 ^a^			
Cognitive	HHCG	0.984	0.337 ^a^	0.00 (0.00, 16.67)	0.143	0.892 ^d^
	Control	0.462	0.796 ^b^			
Social	HHCG	3.347	0.003 ^a,^*	16.67 (0.00, 33.33)	1.903	0.058 ^d^
	Control	1.605	0.135 ^b^			
Global health status	HHCG	0.128	0.098 ^b^	0.00 (−8.33, 8.31)	0.068	0.987 ^d^
	Control	0.118	0.907 ^a^			
**PDI (total)**	HHCG	3.228	0.004 ^a,^*	−9.00 (−17.00, −2.00)	2.470	0.013 ^d,^*
	Control	2.247	0.034 ^a,^*			
Symptom distress	HHCG	3.501	0.002 ^a,^*	−2.33 (−4.80, 0.14)	1.897	0.064 ^c^
	Control	1.960	0.104 ^a^			
Existential distress	HHCG	3.156	0.005 ^a,^*	−3.20 (−6.88, 0.47)	1.774	0.086 ^c^
	Control	2.088	0.048 ^a,^*			
Dependency	HCCG	0.512	0.615 ^a^	0.00 (−2.00, 1.00)	1.396	0.166 ^d^
	Control	0.496	0.312 ^a^			
Social support	HCCG	1.860	0.083 ^a^	−0.08 (−1.65, 1.49)	0.103	0.919 ^c^
	Control	1.690	0.104 ^a^			
Peace of mind	HHCG	1.983	0.047 ^a,^*	−0.10 (−1.71, 1.51)	0.126	0.900 ^c^
	Control	2.850	0.009 ^a,^*			
**Psychological distress**	HHCG	2.686	0.014 ^b,^*	0.00 (−2.00, 0.00)	1.396	0.166 ^d^
	Control	1.127	0.271^a^			

Abbreviations: HHCG = Heart to Heart Card Game. PDI = Score on The Patient Dignity Inventory. ^a^ Paired sample *t* test; ^b^ Wilcoxon Signed Ranks Test; ^c^ Independent sample *t* test; ^d^ Mann–Whitney U; * *p* < 0.05.

## Data Availability

The data presented in this study are available on request from the corresponding authors.

## Supplementary Materials

The following supporting information can be downloaded at: https://www.mdpi.com/article/10.3390/ijerph19106115/s1, Supplementary File S1: Heart to Heart Card Game Intervention; Supplementary File S2. Case A.
