# Supplementary material for: Effects of the Heart to Heart Card Game for Patients with Advanced Cancer Receiving Home-Based Palliative Care: A Clinical Randomized Controlled Trial
Source: Int J Environ Res Public Health. 2022 May 17;19(10):6115. doi: 10.3390/ijerph19106115 (PMC9140332; doi:10.3390/ijerph19106115)
Supplement: Supplementary file 1 [file ijerph-19-06115-s001.zip › Supplementary File S1. Heart to Heart Card Game Intervention.pdf]

Supplementary File S1. Intervention.

The process of Heart to Heart Card Game

**Session**	**Theme**	**Content and Example**
Session 1：Preparation and Introduction	Environmental preparation	■ Create a private and quiet space at patients' homes.<br>■ Require a specific space to place twelve playing cards.<br>■ Only researchers and patients are in the space (The patient's family members may influence the patient's choices).
	Introduce the Heart to Heart Cards to the patients	■ Explain to the patient the origin, composition, and purpose of the Heart to Heart Cards Game.<br>■ Introduce to the patient the principles of the game. Emphasize the principle of voluntary.<br>■ Give the cards to the patient and let the patient know the contents of the card.
	Get permission	■ Ask the patient if he/she would like to continue.<br>■ Introduce the rules of the game if the patient is willing to continue.<br>■ If the patient refuses, introduce the patient share the reason for the refusal.
Session 2：Choosing cards	Chose 12 from 54 cards	■ Let patients choose 12 cards from the deck that was important or fit to themselves most (if the patient do not have a satisfactory card, a Joker card can be used instead) and set the rest of the deck aside.
	Chose 3 from 12 cards	■ Let patients choose the three most important cards from the 12 cards, then sort by importance.
Session 3：End-of-life conversation	Semi-structured interview	■ Patients were asked the following questions to share why they chose these cards:<br>● How do you understand the content on the card?<br>● Why do you think this card (issue) is important to you?<br>● Do you think you have achieved this wish? If not, how do you want to achieve it?

		If yes, do you want to keep the status or still want to do something?<br>● What's you want to tell them or you want them to remember or do?
	Make wish list	■ List the patients' fulfilled and unfulfilled wishes or preferences and mark with "to be completed", "completed' or "maintain" ("to be completed" means a desire to achieve in the future; "completed' or means has been achieved and no further intervention is required; "maintain" means that it has now been implemented but needs the effort to maintain the state)
	Confirm information	■ Summarize the content of the semi-structured interview and confirm them to the patient.
Session 4：<br>Information Translations	Information transfer and Give personalized support	■ Transferred the caregivers about the patient's thoughts and gave advice\support. Including but not limited to the following:<br>● Tell the family members what the want their family members to do.<br>● Provide a means of contact for charities.<br>● Give symptom management and related advice based on patient symptoms.<br>● Help patients coordinate family relationships.<br>● Help patients maintain existing care.
