# Supplementary material for: Effects of the Heart to Heart Card Game for Patients with Advanced Cancer Receiving Home-Based Palliative Care: A Clinical Randomized Controlled Trial
Source: Int J Environ Res Public Health. 2022 May 17;19(10):6115. doi: 10.3390/ijerph19106115 (PMC9140332; doi:10.3390/ijerph19106115)
Supplement: Supplementary file 1 [file ijerph-19-06115-s001.zip › Supplementary File S2. Case A.pdf]

Supplementary File S2

Case A

General information:

Gender: male

Age:62,

Diagnosis: lung cancer

Session 1：Preparation and Introduction

- Setting: Participant played Heart to Heart Cards Game in living room.

Session 2：Chose cards

- Chose 12 from 54 cards

  The following are the results of patient selection:

1. Heart A: I want to maintain me dignity.
2. Diamonds 7: I do/don’t want to die at home. (don’t want to die at home)
3. Spade 6: I want to look nice
4. Heart 8: Don’t put my body in the freezer until 8 hours after I die.
5. Heart K: I want to listen to the music I like.
6. Diamonds 10: I want to be go outside.
7. Diamonds Q: I’d like room with windows.
8. Club A: I don’t want to be a burden to my family.
9. Club 8: I want my family to get along.
10. Club K: I want a chance to help others
11. Spades A: I don’t want to suffer
12. Spades 9: I want to be alert when I die

- Chose 3 from 12 cards

  The following are the results of patient selection:

1. Heart A: I want to maintain me dignity.
2. Diamonds 7: I do/don’t want to die at home. (don’t want to die at home)
3. Spade 6: I want to look nice

Session 3：End-of-life conversation

■ Semi-structured interview

E.g., The participant chose: Heart A: I want to maintain me dignity.

- How do you understand the dignity?

  This participant believed that dignity is that the family needs to listen carefully to the views he presents and specific examples were stated with the guidance of the nurse. Participant put that in this way: *"dignity, for me, I think is, respect me, I hope my family can listen to me carefully…they didn't hear what I said at all. I was talking to the wind…I mentioned many times that I wanted to buy a cemetery, and they just said they knew. They didn't take it seriously."*.

- What's you want to tell your family caregivers or you want them to remember or do most?

  This participant noted that the thing he most wanted his family to do at the moment was to buy a cemetery.

- Why do you think cemetery is important to you?

  The participant said that because he is not a local person, the culture of his hometown emphasizes cemeteries and feng shui.

■ Make wish list

1. I want to maintain me dignity (to be completed).
2. Don't want to die at home (to be completed).
3. I want to look nice (maintain).
4. Don't put my body in the freezer until 8 hours after I die (to be completed).
5. I want to listen to the music I like (maintain).
6. I want to be go outside (to be completed).
7. I'd like room with windows (maintain).
8. I don't want to be a burden to my family (to be completed).
9. I want my family to get along (maintain).
10. I want a chance to help others (to be completed).
11. I don't want to suffer (maintain).
12. I want to be alert when I die (to be completed).

Session 4：Information Translations

Inform family members:

Participants want to participate in discussions of family events and want to buy a cemetery.

Participants hope not to die at home, and not to enter the freezer for eight hours after death, hoping to handle the funeral according to the customs of his hometown.

Participants hoped that even after his death, the family would be able to get together more often.

Participants hope to go downstairs for fresh air.

The participant wishes to download his favorite music to him.

Help the patient adjust the dose of painkiller.
